# An overview of female genital mutilation

**DOI:** 10.4274/tjod.galenos.2019.77854

**Published:** 2019-07-03

**Authors:** Berna Dilbaz, Nuray İflazoğlu, Sıdıka Aydan Tanın

**Affiliations:** 1 University of Health Sciences, Etlik Zübeyde Hanım Gynecological Diseases Training and Research Hospital, Clinic of Obstetrics and Gynecology, Ankara, Turkey

**Keywords:** Female genital mutilation, infibulation, sexual dysfunction

## Abstract

Female genital mutilation (FGM) includes procedures that intentionally alter or cause injury to the female genital organs for non-medical reasons. To present a case of type III FGM corrected by de-infibulation for treatment of sexual dysfunction. A 31-year-old woman who had FGM reporting unconsummated marriage presented to our clinic clinic. The patient had undergone type III FGM at age 7 in her country. Surgical correction was performed. By de-infibulation, the vaginal and urethral orifices were revealed after incision of scar tissue. The World Health Organization classifies FGM in four types. Type III FGM is narrowing of the vaginal orifice with the creation of a covering seal by cutting and appositioning the labia minora and/or the labia majora, with or without excision of the clitoris (infibulation). De-infibulation surgery is recommended for resolving problems related with sexual dysfunction and child-birth.

**PRECIS:** To identify the possible risk factors for postpartum urinary retention.

## Introduction

Female genital mutilation (FGM) includes procedures that intentionally alter or cause injury to the female genital organs for non-medical reasons. The practice, which is also known as female circumcision or female genital cutting, is typically performed by a traditional circumciser using a blade under unsafe conditions, mostly on young girls between infancy and age 15 years. The idea of performing the procedure by medical staff in order to make it safer is condemned because this procedure is internationally accepted as a violation of the human rights of girls and women^(1,2)^. More than 3 million girls are estimated to be at risk each year, and 200 million women living today in 30 countries have undergone the procedures^(1)^. The practice is most common in the western, eastern, and north-eastern regions of Africa, in some countries of the Middle East and Asia, as well as among migrants from these areas. There have been international efforts to persuade practitioners to abandon FGM, and it has been outlawed or restricted in most of the countries in which it occurs.

The procedures are mostly performed on young girls sometime between infancy and adolescence, and occasionally on adult women. The reasons why FGM is performed vary from one region to another. It is often considered as a necessary part of raising a girl, and a way to prepare her for adulthood and marriage. FGM is a social convention (social norm), there is social pressure to comply with the expectations of society, and the need to be accepted socially and the fear of being rejected by the community leads to FGM being performed in these countries. FGM is often motivated by beliefs about what is considered as acceptable sexual behavior^(3)^.

Globalization and immigration has led to the recognition of this problem in countries where FGM is not practiced^(4)^. The European Institute of Gender Equality estimates that 180,000 women and girls are at risk for FGM each year in Europe.

World Health Organization (WHO) classifies this procedure in four types^(4)^.

### WHO classification of female genital mutilation

Type I (clitoridectomy): this is the partial or total removal of the clitoris (a small, sensitive and erectile part of the female genitals), and in very rare cases, only the prepuce (the fold of skin surrounding the clitoris).

Type II (excision): this is the partial or total removal of the clitoris and the labia minora, with or without excision of the labia majora.

Type III (infibulation): this is the narrowing of the vaginal opening through the creation of a covering seal. The seal is formed by cutting and repositioning the labia minora, or labia majora, sometimes through stitching, with or without removal of the clitoris (clitoridectomy).

Type IV: this includes all other harmful procedures to the female genitalia for non-medical purposes, e.g. pricking, piercing, incising, scraping, and cauterizing the genital area.

In some populations, infibulation, which involves cutting the labia and suturing the vulva leaving a small orifice for urination and menstruation, constitutes 15% of cases^(5)^. FGM in the short term may lead to severe complications such as pain, excessive bleeding (hemorrhage), genital tissue swelling, fever, infection, urinary problems (painful urination, urinary tract infections), vaginal problems (discharge, infections), menstrual problems (dysmenorrhea, hematocolpos), sexual and psychological problems, and difficulty in pregnancy and childbirth may arise after the procedure.

Long-term consequences include: urinary problems (painful urination, urinary tract infections; vaginal problems (discharge, itching, bacterial vaginosis and other infections), menstrual problems (e.g. painful menstruations, difficulty in passing menstrual blood), keloid formation on the scar tissue, sexual problems (e.g. pain during intercourse, decreased satisfaction), increased risk of childbirth complications (e.g. perineal tear, difficult or prolonged labor, increased rate of cesarean section and postpartum hemorrhage, increased need for newborn resuscitation), and rarely newborn deaths.

A need for later surgeries may arise due to FGM, especially with FGM procedures that seal or narrow the vaginal opening, which needs to be cut open later to allow for sexual intercourse and childbirth (de-infibulation). Sometimes genital tissue is stitched again several times (re-infibulation), especially after the delivery. Then the woman has to endure repeated opening and closing procedures and thus immediate and long-term risks, psychological problems (e.g. depression, anxiety, post-traumatic stress disorder, low self-esteem), and health complications of FGM are augmented^(6)^.

A patient with type III FGM who presented to our clinic because of sexual dysfunction and underwent de-infibulation surgery is presented.

## Case Report

**Figure 1:**
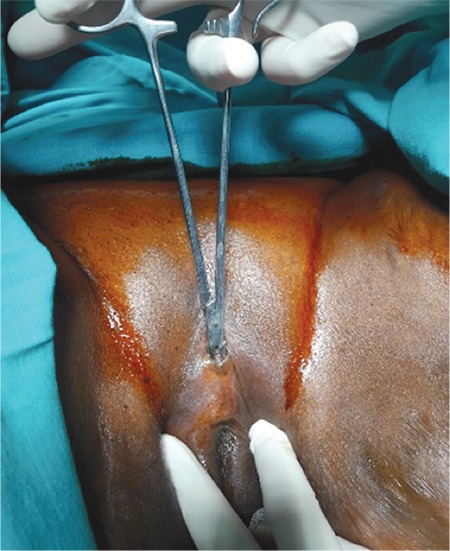
Insertion of Kelly clamp through the orifice and moved caudally in the tunnel-shaped space formed under the fusion line of the scar tissue

Our patient was a 31-year-old, gravida 0 woman with regular menstrual cycles. She had been married for two weeks. She was admitted to our clinic because she was unable to have vaginal sexual intercourse. The patient had undergone genital mutilation at the age of 7 years in Somalia. A gynecologic examination revealed a totally excised labia majora and labia minora and clitoris, and a single orifice was observed in the perineum (Figure 1).

**Figure 2:**
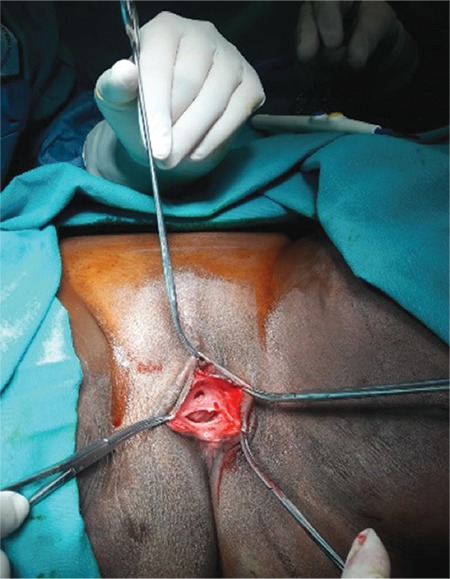
After the scar tissue was excised urethral orifice, vaginal introitus and an intact hymenal membrane were observed

**Figure 3:**
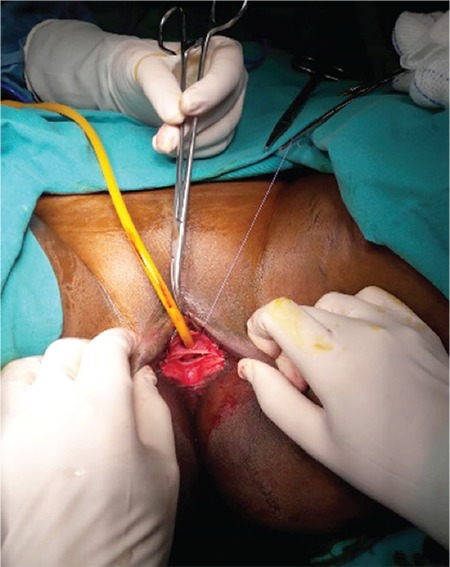
Restoration and visualization of the vaginal introitus and urethral orifice

Detailed counselling was conducted by the gynecologist and a written signed consent was obtained that covered an explanation of the de-infibulation procedure, and the patient also gave consent for the publication of the case.  The de-infibulation procedure was performed under anesthesia. The patient was prepared in the lithotomy position under sterile conditions. A Kelly clamp was gently inserted through the orifice and moved caudally into the tunnel-shaped space formed under the fusion line of the scar tissue. The scar tissue was then excised medially under the guidance of the Kelly clamp. The urethral orifice was observed at the upper part of the vaginal introitus and the hymenal membrane was found to be intact (Figure 2). The separated ends of the remains of the labia were repaired by suturing. Subsequently, bladder catheterization was performed in order to control the patency of the urethra, which showed a normal urethra (Figure 3). At the end of the procedure, a dressing with estriol cream and nitrofurazone was applied on the incision site in order to enhance epithelization. The patient had daily cleaning and dressing applications and was discharged uneventfully at day 3 postoperatively. The couple upon discharge was referred to the sexual dysfunction clinic of the hospital. During the 2-month follow-up after the surgery, the patient had a full recovery and was able to perform vaginal intercourse uneventfully.

## Discussion

Girls ranging from newborn up to the adolescent period and rarely adult women are at risk for FGM in societies and communities where FGM is a tradition. Cultural and social reasons of FGM vary according to region. The reasons include: religion, fear of exclusion by society, protection of virginity before marriage, reduction of extramarital sexual unity, being seen as a part of the upbringing of girls or as a preparation for marriage and adulthood, perception of beauty and cleansing, and a necessity for being a woman.

For many years, international efforts have been made to ensure that those who practice FGM abandon this procedure^(1)^. As a result of these efforts, implementation is prohibited or restricted in most countries; however, these laws are not fully implemented. The short-term complications of FGM range from pain, infection, and bleeding to death due to these complications. HIV and tetanus infections are the most serious infections that might arise after FGM. These infections have a higher incidence in women with type II FGM^(7)^. Reyners et al.,^(8)^ reported an estimated mortality of 1 in 500 circumcisions. In countries where FGM is not a tradition or a norm, acute complications of FGM can be very rarely seen, but medical workers may still encounter women with FGM-related long-term health problems ranging from urogenital disorders to sexual and mental health problems because of immigration from the countries where FGM is a well-accepted practice among the community members. Difficulty during the first vaginal penetration is very common especially in women with infibulation because the first vaginal sexual act results in pain and sometimes requires a surgical intervention or results in perineal tearing^(9)^. De-infibulation restores urinary and vaginal function, but these women need further counselling and support due to the psychological effects of this traumatizing experience^(10,11)^.
